# Cross-lagged analysis of rumination and social anxiety among Chinese college students

**DOI:** 10.1186/s40359-023-01515-6

**Published:** 2024-01-16

**Authors:** Peibo Wu, Keyan Cao, Wenjing Feng, Shuai Lv

**Affiliations:** 1 Institute of Education, Xiamen University, Xiamen, People’s Republic of China; 2 Zhong yuan Institute of Science And Technology, Zhengzhou, People’s Republic of China; 3 School of Modern Logistics, Qingdao Harbour Vocational and Technical College, Qingdao, People’s Republic of China; 4 Shenzhen Polytechnic University, Shenzhen, People’s Republic of China

**Keywords:** College students, Rumination, Social anxiety, Cross-lagged model, Longitudinal study

## Abstract

**Background:**

Social anxiety, which is widely prevalent among Chinese college students, poses a significant barrier to their holistic psychological and physiological development. Although numerous cross-sectional studies have examined the relationship between rumination and social anxiety, there is still a gap in understanding their interplay over time. This longitudinal study aimed to explore and analyze the intricate interrelations between these two factors, with the ultimate goal of informing the development of effective mental health education interventions for university students.

**Methods:**

Using the Ruminative Responses Scale (RRS) and the Interaction Anxiousness Scale (IAS), a two-stage longitudinal follow-up study of 392 college students from three universities in Henan Province was conducted over a six-month period (October 2022 to March 2023) using a cross-lagged model to explore the correlation between rumination and social anxiety. The results of the correlation analysis showed that rumination was positively associated with social anxiety at both time points (*r* = 0.18,0.12, *p* < 0.01).

**Results:**

Cross-lagged regression analyses revealed that the predictive effect of the first measure (T1) rumination on the second measure (T2) rumination was statistically significant (β = 0.32, *p* < 0.001). The predictive effect of T1 social anxiety on T2 social anxiety was statistically significant (β = 0.65, *p* < 0.001), the predictive effect of T1 rumination on T2 social anxiety was statistically significant (β = 0.33, *p* < 0.001), and the prediction of T1 social anxiety on T2 rumination was statistically significant (β = 0.28, *p* < 0.001).

**Conclusion:**

College students’ rumination and social anxiety are mutually predictive of each other, and interventions by educators in either of these areas have the potential to interrupt the vicious cycle between ruminant thinking and social anxiety.

**Supplementary Information:**

The online version contains supplementary material available at 10.1186/s40359-023-01515-6.

## Introduction

Social anxiety is a maladaptive emotional experience characterized by apprehension, fear, and worry in response to real or imagined social interactions and situations [1]. Research indicates that college students are a high-risk population for social anxiety, with a lifetime prevalence rate exceeding 12% [2]. Furthermore, there is no significant gender difference in the patterns of onset [3–5]. Social anxiety frequently co-occurs with various psychological disorders such as depression and avoidant personality disorder [6], with studies revealing that 48% of social anxiety sufferers also have comorbid depression [2]. Particularly, the outbreak of the COVID-19 pandemic led to the implementation of isolation measures in multiple countries, severely restricting interpersonal interactions among college students [7]. This further exacerbated social anxiety within this demographic, resulting in increased levels of depression, loneliness, and related issues [3, 8].

As a prevalent psychological disorder among college students, social anxiety significantly affects their academic and personal lives [9]. socially anxious college students suffer not only from academic maladjustment due to their inability to interact with teachers normally [10–12] but also from academic burnout and academic dishonesty [13, 14]. Social anxiety can even lead to depression or suicidal behavior in college students [2, 12]. Given the substantial impact of social anxiety on college students’ development, it is essential to explore its contributing factors and related issues. These endeavors are not only of paramount importance for the healthy growth of college students but also provide valuable strategies for educators and researchers to improve educational practices.

Numerous studies have discussed the influencing factors of social anxiety in college students, among which rumination is a vulnerability factor causing this problem [15–17]. Rumination is defined by Nolen-Hoeksema, referring to individuals continuously dwelling on the causes and consequences of negative life events without taking action [18]. Individuals who ruminate often exhibit a cognitive bias in information processing, leading them to concentrate on and recall negative information rather than positive information. Therefore, rumination further exacerbates negative emotions such as anxiety and depression in individuals [19]. Furthermore, scholars have pointed out that girls are more prone to rumination than boys and are also more likely to experience anxiety and depressive emotions [15, 20]. The cognitive model of social anxiety suggests that rumination involves repeatedly contemplating adverse consequences in social situations with a negative bias, and individuals engage in rumination both before and after social interactions [21]. Rumination perpetuates a vicious cycle of social anxiety in college students [17, 22]. Although different scholars may have varying definitions of rumination, overall, existing research predominantly supports that rumination is a negative cognitive process characterized by the repeated contemplation of the causes and outcomes of events with a negative bias, without taking action [14, 17, 23]. Studies have indicated that individuals who remain in a prolonged and extensive state of rumination after experiencing negative social life events may develop social anxiety or even depressive states [19, 24]. The academic community has demonstrated the close relationship between rumination and social anxiety [22, 25, 26]. Rumination not only leads to social anxiety, but individuals with social anxiety, unable to meet the expectations of others, develop negative self-awareness and may become trapped in rumination [17, 27]. Although the link between rumination and social anxiety is recognized, further empirical research is needed to draw definitive conclusions, especially in exploring the mutual predictive relationship between rumination and social anxiety.

However, there are inconsistencies in the empirical research findings. Some studies suggest that rumination is a risk factor for social anxiety [18, 19, 23], while others propose that rumination is a negative consequence of social anxiety [2, 25]. Since such studies often rely on cross-sectional data, determining the directional relationship between these factors becomes challenging. The bidirectional predictive relationship between rumination and social anxiety in the temporal dimension merits empirical investigation. It has been indicated that both rumination and social anxiety undergo changes with age and contextual shifts among adolescents [22, 28]. Therefore, it is necessary to conduct longitudinal tracking studies to explore whether there is a reciprocal predictive relationship between rumination and social anxiety.

There are three views of the relationship between rumination and social anxiety. First, it is believed that rumination can positively predict social anxiety [23, 26]. According to the cognitive model of social anxiety, rumination plays a crucial role in perpetuating social anxiety [22]. Essentially, individuals are prone to exhibit avoidant behaviors in social interactions when they harbor negative self-cognition and self-beliefs regarding past or anticipated social scenarios [6, 29, 30]. It is proposed herein that individuals experiencing negative cognition tend to perceive others as potential threats and may engage in risk assessments 23of ambiguous interpersonal situations. In addition, feeling afraid of the negative effects caused by interpersonal communication, they may become socially anxious [31, 32]. Other studies show that people opt to have negative rumination on themselves and that environments are more likely to evoke anxious emotions [16, 17, 26, 33]. Similarly, rumination can not only directly predict the manifestation of social anxiety but also indirectly forecast its progression by activating intervening variables, such as negative self-cognition and loneliness [34–36]. These results have unanimously confirmed the influence of rumination on social anxiety [37, 38].

Second, rumination is considered a negative consequence of social anxiety [39, 40]. According to Schwarz and Clore’s feelings-as-information theory, emotional states affect people’s information processing methods. If an individual is in a negative emotional state, he or she will initiate the negative attribution mode and maintain consistency between the attribution styles and the negative emotion [41]. As presumed, socially anxious individuals universally concentrate on negative life events [42]. They may perceive normal interactions as a distressing thing and thus feel negative about communication. Furthermore, to make emotions concordant with cognition, they will reflect on and appraise themselves insistently rather than take steps to address issues [43, 44]. Similar studies note that individuals, feeling socially anxious, often have impaired control over attentiveness. That is, they are unable to distract their attention from repeated negative thoughts but only think about things passively [2, 21, 23]. Likewise, it has been demonstrated that the more serious social anxiety is, the more time people are willing to spend alone. Meanwhile, immersion in negative evaluations of themselves and external events further aggravates rumination [33, 45]. Therefore, social anxiety is an important antecedent variable for the formation of rumination [39].

Third, it is proposed that rumination and social anxiety are mutually predictive of each other. In the integrated model constructed by Dignath et al. for cognitive conflicts and negative feelings, the former can induce the latter. People with these feelings tend to center on pessimistic thoughts and process negative messages, which interact to form a dynamic circulatory system [46, 47]. This work reveals that individuals who recognize the external world in a negative manner will feel socially anxious about interactions in reality because of their excessive attention to psychological activities, which triggers the top-down cognitive processing of negativity. In other words, anxious individuals prefer to process anxious stimuli, which, in turn, reactivate their anxious feelings to create a vicious cycle [48]. Moreover, cognitive neuroscience also provides evidence of the interrelationship between emotions and cognition, both of which originate from the same cortical system [49]. Therefore, it can be deduced that rumination and social anxiety may be mutually predictive.

Most of the abovementioned studies exploring the relationship between rumination and social anxiety are based on cross-sectional designs. These results have certain limitations in revealing the relationships between variables and cannot depict the mutual predictive relationship between rumination and social anxiety over time. If rumination and socialization are predictive of each other, the cumulative effect of social anxiety disorders, even to the point of severe depression and avoidance behaviors. This also poses challenges for the implementation of mental health education in universities. Therefore, this study intends to use a cross-lagged analysis method to conduct a 6-month longitudinal tracking study at two different time points, namely, October 2022 (T1, the same below) and March 2023 (T2, the same below), aiming to investigate the mutual predictive relationship between rumination and social anxiety in terms of temporal changes. This study aims to provide more substantial evidence for the relationship between these two variables. Based on the a forementioned discussions, this study hypothesizes that there is a mutual predictive relationship between rumination and social anxiety among Chinese college students in the temporal dimension.

## Methods

### Participants

In this study, we employed a whole-group sampling approach to recruit first-year undergraduate students from the Education Colleges of three private Universities in Henan Province who are part of the 2022 cohort. These universities are primarily focused on teaching, and each institution has a student population exceeding 12,000. The study was conducted at two different time points, October 2022 (Time 1, below) and March 2023 (Time 2, below), and we constructed a cross-lagged model to explore the mutual predictive relationship between rumination and social anxiety among Chinese college students through a 6-month follow-up study. There were 589 volunteers in Phase T1, 471 (79.97%) female and 118 (20.03%) male students, whose mean age was 18.18 years and SD = 1.13 years. There were 417 volunteers in Phase T2, 336 (79.97%) female and 81 (19.42%) male students, the participant attrition rate was 29.2%. After the data were matched twice using the students’ school numbers, 392 valid paired questionnaires were finally matched for pre- and post-measurement data. Of these, 63 (16.07%) were completed by male students and 329 (83.93%) by female students. It was worth noting that, although longitudinal tracking studies ideally involved the same cohort of participants, this study adhered to the principle of voluntariness, resulting in a certain degree of sample attrition.

This study primarily collected data through the Sojump platform, an online survey platform with high recognition in China. This study followed the principles of the Research Ethics Committee of the research institute and was approved by the principals of the participating schools. The participants were informed of the purpose of the study, the nature of voluntary participation, and how to withdraw from the survey. Informed consent was obtained from all of the participants prior to their participation in the study.

### Measures

#### Ruminative responses scale-RRS

We used the Chinese version of the Ruminative Responses Scale (RRS), developed by Susan Nolen-Hoeksema, which ultimately included 22 items, an example of which is “If I can’t stop thinking about this, then I can’t get on with the task at hand”. The scale is divided into three dimensions, symptomatic rumination, obsessive thinking and reflective rumination, with each entry option set on a 4-point Likert scale from “1= absolutely disagree” to “4= absolutely agree”. The higher the total score is, the more severe the rumination. Research has confirmed that the RRS has good reliability and validity in the Chinese university population [50]. In this study, Cronbach’s alpha coefficients of the RRS were 0.96 and 0.92, respectively. The structural validity of the first measurement was χ2/df = 2.878, CFI = 0.914, ILI = 0.915, TFI = 0.900, RMSEA = 0.070, SRMR = 0.023; the structural validity of the second measurement was χ2/df = 2.606, CFI = 0.915, IFI = 0.916, TLI = 0.900 RMSEA = 0.064, SRMR = 0.022.

#### Interaction anxiousness scale-IAS

The Interaction Anxiousness Scale (IAS) was developed by Leary (1983) and revised by Chinese scholar Wang Xiangdong et al. [51, 52]. The scale consists of 15 items; an example item is “I feel nervous even at informal gatherings”. Questions 3, 6, 10 and 15 of the scale are scored inversely so that they are processed positively, and the final scores are summed, with higher scores indicating higher levels of subjective anxiety experienced in social situations. Each item was scored on a 5-point Likert scale from “1 = not at all” to “5 = completely correct”. Cronbach’s alpha coefficients for the questionnaires were 0.84 at Time 1 and 0.78 at Time 2. The structural validity of the first measurement was χ^2^/df = 2.878, CFI = 0.925, IFI = 0.926, TLI = 0.900, RMSEA = 0.077, SRMR = 0.062; the structural validity of the second measurement was χ^2^/df = 2.909, CFI = 0.927, IFI =0.928, TLI = 0.907, RMSEA = 0.064, SRMR = 0.054.

### Procedure

First, the common method biases of our data were examined by one-way analysis of variance. Second, we used SPSS 24.0 software to explore rumination and social anxiety at both the T1 and T2 time points based on descriptive statistical analysis (means and standard deviations) and Pearson correlation coefficients. Third, a cross-lagged model was created for two variables using AMOS 24.0 to further verify their relations. Herein, we not only reviewed the hypothetical model’ fitness to the data but also tested four nested models (M1-M4). Therefore, the baseline model (M1), which only included the autoregression, was used to measure the lateral stability between rumination and social anxiety and the fluctuation at both time points [53]. On this basis, we built M2 and M3, of which M3 was added with a cross-lagged path that was similar but different to that of M2: the path from social anxiety at T1 to rumination at T2. The r) that included all the paths in the above models was compared with M1, M2, and M3 to determine the final model that best fitted the data, i.e., the possible causal relationship.

## Results

In this study, we gathered data concerning college students’ rumination and social anxiety using self-report methods, potentially introducing certain common method biases. Following the recommendations by Podsakoff et al. (2003), we scrutinized the common method biases inherent in the two datasets using Harman’s single-factor test to maintain the validity of the study [54]. The results showed that in the two measurements, the first factor could only explain the amount of variability of 27.139 and 27.309%, smaller than the critical value of 40%, which indicated that no obvious biases were detected in our data.

Table 1 lists the overall item means, standard deviations, and correlation matrices of both rumination and social anxiety. The results revealed a slight increase in the overall item means of rumination and social anxiety from T1 to T2. However, the effect sizes for both cases (Cohen’s d = 0.06 and Cohen’s d = 0.08) were both below 0.2, indicating a small effect size according to Cohen (1988) [55]. The differences between the two time points were not substantial. There was a significant positive correlation between rumination scores at each time point (*r* = 0.32, *p* < 0.01), and similarly, social anxiety scores also showed significant positive correlations between each time point (*r* = 0.59, *p* < 0.01). In terms of simultaneous correlation, a significant positive correlation was found between rumination and social anxiety at both the T1 and T2 time points (*r* = 0.18 and 0.12, *p* < 0.01). In terms of ephemeral correlation, rumination at T1 was significantly positively associated with social anxiety at T2 (*r* = 0.40, *p* < 0.01), and rumination at T2 was significantly positively associated with social anxiety at T1 (*r* = 0.27, *p* < 0.01). In summary, the above results are in line with the underlying assumptions for conducting cross-lagged analysis.

**Table 1:** Means, standard deviations, and correlations among the main measures (*N* = 392)

Variable	M	SD	t	Cohen’s d	1	2	3
1.T1 Rumination	2.27	0.21	0.40	0.06	1		
2.T2 Rumination	2.28	0.15			0.32^**^	1	
3.T1 Social Anxiety	3.31	0.21	1.15	0.08	0.18^**^	0.27^**^	1
4.T2 Social Anxiety	3.33	0.30			0.40^**^	0.12^*^	0.59^**^

T1, T2, =Time 1, Time 2;^**^ *p* < 0.01, ^*^ *p* < 0.05

**Fig. 1:**
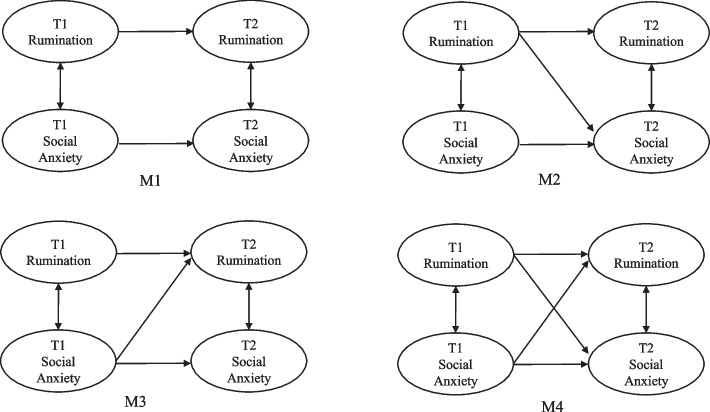
Nested models of the relationship between Rumination and Social Anxiety

To more deeply explore the reciprocal predictive relationship between college students’ rumination and social anxiety, we followed the advice of Martens and Haase (2006) and performed a cross-lagged analysis on two groups of measured data [53]. Prior to this, their interrelationship was first discussed by inspecting the relations and comparing the fit indices of four theoretical models (Fig. 1). M1 is the baseline model; M4 is the full model. Table 2 shows these indices and the chi-square difference between each competitive model (M1, M2, and M3) and the full Model (M4), with the observation that M4 had a better fit index than the other three and showed an obvious chi-square difference with M1 (Δχ^2^ = 85.369, Δdf = 2, *p* < 0.001), M2 (Δχ^2^ = 22.040, Δdf = 1, *p* < 0.001), and M3 (Δχ^2^ = 46.309, Δdf = 1, *p* < 0.001). The model with the best fitting result is the final model (Table 2).

**Table 2:** The goodness-of-fit statistics for the nested models

Model	χ^2^	Df	CFI	TLI	SRMR	RMSEA	Model comparisons	Δχ^2^	Δdf	*p*
M1	210.974	50	0.93	0.90	0.16	0.09	M4 vs.M1	85.369	2	<0.001
M2	147.645	49	0.96	0.94	0.07	0.08	M4 vs.M2	22.040	1	<0.001
M3	171.914	49	0.95	0.93	0.08	0.07	M4 vs.M3	46.309	1	<0.001
M4	125.605	48	0.97	0.95	0.04	0.06				

**Fig. 2:**
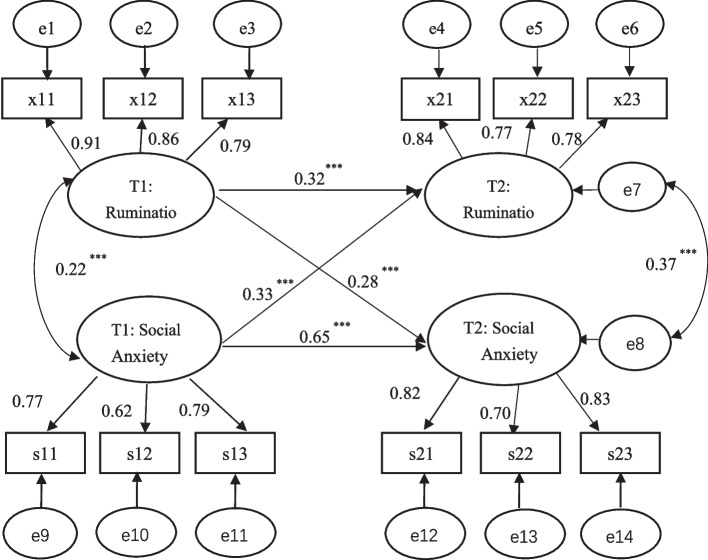
Cross-lagged model of Rumination and Social Anxiety

We constructed a cross-lagged model using AMOS 24.0 and analyzed its fitting degree to the mutuality of rumination and social anxiety. As suggested by Byrne (2010), the fit indices for model evaluation should meet the following requirements: CFI > 0.90, TLI > 0.90, RMSEA < 0.08, and SRMR < 0.08 [56]. The final model is plotted in Fig. 2, where χ^2^/df = 2.62, CFI = 0.97, TLI = 0.95, SRMR = 0.04, and RMSEA = 0.06, showing a good fitting result. Moreover, rumination and social anxiety were extremely stable from T1 to T2, with normalized autoregressive path coefficients of 0.32 (*p* < 0.001) and 0.65 (*p* < 0.001), respectively. In cross-prediction paths, rumination at T1 prominently predicted social anxiety at T2 (β = 0.28, *p* < 0.001), and social anxiety at T1 notably forecasted rumination at T2 (β = 0.33, *p* < 0.001) (Fig. 2).

On this basis, we grouped the participants by gender to validate whether there were gender differences in our cross-lagged model. First, an unrestricted model was built so that all parameters could be freely estimated between genders. Next, a model with identical weight structures was set up to assume the equality of autoregressive and cross-lagged regressive path coefficients between genders. The chi-square test suggested that both models were markedly influenced by equal restricted paths (Δχ^2^ = 12.043, Δ *p* > 0.05), signifying that there was no significant difference in gender in the cross-lagged model for Chinese college students’ rumination and social anxiety (For specific data analysis results, please refer to the Supplementary Material Table s1 and Table s2).

In summary, the results of our cross-lagged model provide support for the mutual prediction and stable interaction of rumination and social anxiety among college students.

## Discussion

Previous studies on rumination and social anxiety of college students have mostly adopted cross-sectional designs, which makes it difficult to accurately reveal the direction of interaction between the two, and it is difficult to provide evidence support for the conflicting theoretical views. This study takes Chinese college students as the object, adopts the longitudinal tracking design with an interval of 6 months, and reveals the mutual predictive relationship between rumination and social anxiety of college students in the time dimension through data at 2 time points.

The results of the correlation analysis show that Chinese college students’ rumination and social anxiety are significantly positively correlated between the two measurements, which is consistent with existing research findings. It has been found that those in a negative emotional state tend to engage in risk assessments of ambiguous interpersonal situations and others around them, which leads to social anxiety. Additionally, individuals with social anxiety are prone to negative self-evaluation and external event appraisal, resulting in rumination [31, 32, 43]. The current findings also indicate that the correlation coefficient at T1 is slightly lower than that at T2. We speculate that this might be attributed to the fact that, after experiencing intense competition in the college entrance examination (gaokao), newly enrolled students engage in implicit social comparisons among peers upon entering the university campus. This might lead them to have intensified negative self-evaluation and self-image when interacting with others, which generates social anxiety [2, 57, 58]. However, as time progresses and new students gradually adapt to university life, some of them cease to imagine normal interpersonal interactions as negative experiences. The dynamic cycle between negative cognition (rumination) and negative emotions (social anxiety) weakens. Therefore, it is essential for university educators to focus on the mental well-being of new college students and enhance their adaptive capacity.

In autoregressive paths, rumination and social anxiety at T1 could remarkably predict rumination and social anxiety at T2, which implied their effect on risk accumulation in the time dimension. Social anxiety and rumination, which are prevalent dynamic emotional states among adolescents, should be emphasized and addressed with interventions in a timely manner to avoid the harm caused by their risk factors. On the contrary, neglecting the risk accumulation phenomenon between rumination and social anxiety in college students will further exacerbate their social anxiety disorders and may even lead to the development of depression or avoidant personality disorders [28, 59]. Additionally, in the key stage of cognitive development and character shaping of college students, it is necessary to adopt mindfulness therapies to promptly break the vicious cycle of rumination and social anxiety [28, 60]. Otherwise, such psychological problems cannot be corrected easily or even cannot be fully treated [28].

The cross-lagged analysis revealed that rumination at T1 significantly predicted social anxiety at T2 (β = *0.28*, *p* < 0.001), and conversely, social anxiety at T1 significantly predicted rumination at T2 (β = 0.*33, p* < 0.001). There is an interactive predictive relationship between the two variables. First, these results are in line with the third point of view and verify or provide preliminary support for the integrated model for cognition and emotion [46, 47]. This might be because in China, college students are influenced by the traditional Confucian culture of “self-reflection,” which could lead them to frequently engage in rumination. However, excessive negative self-reflection could result in individuals becoming trapped in negative self-memory, thereby generating social anxiety during interpersonal interactions. Those with social anxiety tend to immerse themselves in the negative self-image memory of social situations, constantly recalling unfavorable events from social interactions. This further escalates the frequency of negative self-reflection, intensifies social anxiety, and forms a wretched cycle between rumination and social anxiety [2, 22, 28]. Previous studies have validated that individuals who habitually engage in negative cognitive processing styles usually have a distorted cognition of both themselves and the world [22, 23, 58], making them more anxious about interpersonal communication [61]. In addition, people who are feeling downcast are inclined to keep their mind on negative and shameful autobiographical memories due to close attention to threat-related stimuli. Thus, they cannot relieve anxiety through the social support of others, which eventually brings about rumination [62–64].

In this study, through a multi-group analysis, we found that there were no significant gender differences in the cross-lagged regression path coefficients between rumination and social anxiety. This implies that the impact of rumination on social anxiety and vice versa is similar across genders. Our results align with existing research that indicates minimal gender differences in the correlation between rumination and anxiety [65]. This may be associated with China’s historical one-child policy. Present-day Chinese college students, whether male or female, often grow up in single-child families, leading to a common lack of interpersonal skills and strategies. They tend to focus more on their self-image in social interactions, often becoming overly concerned about whether their self-image meets others’ expectations, resulting in social anxiety [25, 30]. Conversely, underperforming in social situations can reinforce negative self-image, perpetuating negative interpretations of social scenarios. Furthermore, the absence of gender differences in the relationship between rumination and social anxiety aligns with the gender similarity hypothesis, suggesting that men and women exhibit similarity in most psychological variables [66]. This implies that personal traits have a limited influence on the interaction between rumination and social anxiety, highlighting the cross-gender stability in the relationship between these two factors. In essence, the impact of rumination and social anxiety appears to be consistent across different gender groups. Studies have suggested that Asian adolescents are affected by the traditional culture of suppressing individuality and maintaining social harmony, so they are more susceptible to self-reflection and social anxiety. Conversely, in individualistic cultures where personal uniqueness and standing out are valued, social anxiety might be interpreted as a lack of confidence, resulting in fewer cases diagnosed as social anxiety [67, 68]. This finding provides guidance for psychological health education among Chinese college students. Interventions targeting either rumination or social anxiety could interrupt the cycle, thereby enhancing college students’ psychological well-being.

### Limitations and future directions

Despite the important findings of this study, there are some limitations. First, the primary participants in this study were first-year students in the Education Colleges of three private undergraduate universities in Henan Province. Although the statistical results indicated the absence of common method bias issues, it should be noted that the data exhibited a relatively homogeneous nature. Caution was advised when extending the findings to other demographic groups. Additionally, social anxiety among college students may have been influenced by unrelated variables, such as the adjustment period during their freshman year. Therefore, future studies should consider expanding the sample size and increasing the heterogeneity of the sample by including students from different provinces and different grades, It was essential to control for the influence of unrelated variables and conduct further validation of our findings within college student populations of different academic years. Second, the longitudinal design used in this study avoids the pitfalls of cross-sectional studies to a certain extent, but the data used in this study came from two measurement time points, which only revealed the direction of the effect between the two and did not reveal the dynamic relationship between rumination and social anxiety. Future research could employ more measurement time points, such as by continuously tracking the levels of rumination and social anxiety among college freshmen. This would facilitate a more in-depth exploration of the dynamic relationship between these two variables. Moreover, while this study explored the mutual predictive relationship between rumination and social anxiety through a cross-lagged model, the causal relationship between rumination and social anxiety had not been definitively established. Future research could have investigated this causal relationship between rumination and social anxiety using experimental designs. Third, it should be noted that this study employed a Depressive Rumination Scale, which might have magnified the association between rumination and negative emotions. In future studies, selecting a more suitable rumination scale based on a refined conceptualization of rumination could have yielded more accurate results in relation to social anxiety. Lastly, although the analytical framework assumed stationarity, the study was conducted during the outbreak of COVID-19 in China, which occurred between the two survey periods. Since exposure to COVID-19 (i.e., infection) and exposure to COVID-related publicity were not measured, it is not possible to determine how these factors may have influenced the findings. This represents a significant limitation of our research design, and future studies should take into account the potential impact of these unmeasured variables on the outcomes.

## Conclusions

This study used cross-lagged regression analysis to investigate the reciprocal predictive relationship between rumination and social anxiety among Chinese college students. Our results showed that (1) rumination and social anxiety at the time of enrollment predicted rumination and social anxiety 6 months later, and (2) cross-lagged regression analysis further verified that rumination at the time of enrollment significantly predicted social anxiety 6 months later and that social anxiety at the time of enrollment also predicted rumination. The regression analysis further confirmed that rumination was a significant predictor of social anxiety 6 months later. The results can help educators intervene in college students’ psychological problems and thus improve their mental health reciprocal predictive relationship.

### Supplementary Information

**Additional file 1: Table s1.** Multi-Cluster Analysis Adaptation Table.

**Additional file 2: Table s2. **Invariance Test Sheet.

## Data Availability

The original data of this study have been uploaded to https://figshare.com/ and have generated a Digital Object Identifier (DOI): 10.6084/m9.figshare.24629745. We welcome researchers who are interested in this study to join us for a more in-depth analysis of the research results.
