# Supplementary material for: Cross-lagged analysis of rumination and social anxiety among Chinese college students
Source: BMC Psychol. 2024 Jan 16;12:28. doi: 10.1186/s40359-023-01515-6 (PMC10792811; doi:10.1186/s40359-023-01515-6)
Supplement: Supplementary file 1 — Additional file 1: Table s1. Multi-Cluster Analysis Adaptation Table. [file 40359_2023_1515_MOESM1_ESM.pdf]

Table s1 Multi-Cluster Analysis Adaptation Table

Model	CMIN	DF	P	CMIN/DF	NFI	RFI	IFI	TLI	CFI	RMSEA	AIC	ECVI
Unconstrained	179.797	98	0	1.835	0.925	0.898	0.964	0.951	0.964	0.046	295.797	0.758
Measurement weights	191.84	106	0	1.81	0.920	0.900	0.962	0.953	0.962	0.046	291.84	0.748
Structural weights	202.404	110	0	1.84	0.915	0.898	0.959	0.951	0.959	0.046	294.404	0.755
Structural covariances	203.049	113	0	1.797	0.915	0.901	0.96	0.953	0.96	0.045	289.049	0.741
Structural residuals	215.741	115	0	1.876	0.910	0.896	0.956	0.949	0.955	0.047	297.741	0.763

The results from Table S1 indicate that:

Unconstrained Model: The CMIN value is 179.797 with a DF of 98, indicating a good model fit (CMIN/DF = 1.835). The values of NFI, RFI, IFI, TLI, and CFI are close to or exceed 0.9, demonstrating a good fit of the model.

Measurement Weights Model: The fit indices are close to those of the Unconstrained model, showing similar model applicability.

Structural Weights Model: The CMIN/DF ratio and fit indices indicate a good overall fit of the model.

Structural Covariances Model: The overall fit of the model is similar to the other models.

Structural Residuals Model: The CMIN/DF is slightly higher, but other fit indices still show that the model fits well.

These results support the effectiveness of our research design and the reliability of our data analysis, providing a solid statistical foundation for the conclusions of our study.
