# Supplementary material for: Cross-lagged analysis of rumination and social anxiety among Chinese college students
Source: BMC Psychol. 2024 Jan 16;12:28. doi: 10.1186/s40359-023-01515-6 (PMC10792811; doi:10.1186/s40359-023-01515-6)
Supplement: Supplementary file 2 — Additional file 2: Table s2. Invariance Test Sheet. [file 40359_2023_1515_MOESM2_ESM.pdf]

Table s2 Invariance Test Sheet

Model	ΔCMIN	ΔDF	ΔP	ΔNFI	ΔRFI	ΔIFI	ΔTLI	ΔCFI
Measurement weights	12.043	8	0.149296	-0.005	0.002	-0.002	0.002	-0.002
Structural covariances	22.607	15	0.092848	-0.01	0.003	-0.004	0.002	-0.004
Structural residuals	12.692	17	0.756548	-0.015	-0.002	-0.008	-0.002	-0.009

Based on the results of Table S2:

Measurement Weights: ΔCMIN is 12.043, ΔDF is 8, and the ΔP value is 0.149296. This indicates that there are no significant gender differences in the measurement weights.

Structural Covariances: ΔCMIN is 22.607, ΔDF is 15, and the ΔP value is 0.092848. This suggests that gender may have some impact on structural covariances, but the difference is not highly significant.

Structural Residuals: ΔCMIN is 12.692, ΔDF is 17, and the ΔP value is 0.756548. This indicates that there are no significant gender differences in structural residuals.

In summary, the results of Table S2 indicate that while there may be some impact of gender in certain model aspects, overall, the gender differences are not significant. This suggests a certain level of stability in the applicability of these models when considering gender factors.
